# Transmission Ecology of Sin Nombre Hantavirus in Naturally Infected North American Deermouse Populations in Outdoor Enclosures

**DOI:** 10.1371/journal.pone.0047731

**Published:** 2012-10-26

**Authors:** Karoun H. Bagamian, Jonathan S. Towner, Amy J. Kuenzi, Richard J. Douglass, Pierre E. Rollin, Lance A. Waller, James N. Mills

**Affiliations:** 1 Population Biology, Ecology and Evolution Program, Emory University, Atlanta, Georgia, United States of America; 2 Viral Special Pathogens Branch, National Center for Emerging and Zoonotic Infectious Diseases, Centers for Disease Control and Prevention, Atlanta, Georgia, United States of America; 3 Department of Biology, Montana Tech, University of Montana, Butte, Montana, United States of America; 4 Department of Biostatistics, Rollins School of Public Health, Emory University, Atlanta, Georgia, United States of America; The University of Texas Medical Branch, United States of America

## Abstract

Sin Nombre hantavirus (SNV), hosted by the North American deermouse (*Peromyscus maniculatus*), causes hantavirus pulmonary syndrome (HPS) in North America. Most transmission studies in the host were conducted under artificial conditions, or extrapolated information from mark-recapture data. Previous studies using experimentally infected deermice were unable to demonstrate SNV transmission. We explored SNV transmission in outdoor enclosures using naturally infected deermice. Deermice acquiring SNV in enclosures had detectable viral RNA in blood throughout the acute phase of infection and acquired significantly more new wounds (indicating aggressive encounters) than uninfected deermice. Naturally-infected wild deermice had a highly variable antibody response to infection, and levels of viral RNA sustained in blood varied as much as 100-fold, even in individuals infected with identical strains of virus. Deermice that infected other susceptible individuals tended to have a higher viral RNA load than those that did not infect other deermice. Our study is a first step in exploring the transmission ecology of SNV infection in deermice and provides new knowledge about the factors contributing to the increase of the prevalence of a zoonotic pathogen in its reservoir host and to changes in the risk of HPS to human populations. The techniques pioneered in this study have implications for a wide range of zoonotic disease studies.

## Introduction

Recognition that most emerging infectious diseases are zoonotic [1] has led to increased investigation of wildlife host-pathogen systems designed to characterize pathogens, identify hosts, and describe environmental factors associated with transmission, in order to develop predictive tools and inform control and prevention policies. For example, after a highly fatal outbreak of hantavirus pulmonary syndrome (HPS) in the southwestern USA in 1993, an interdisciplinary team identified a novel hantavirus, Sin Nombre hantavirus (SNV), as the causative agent and the North American deermouse (*Peromyscus maniculatus;* hereafter referred to as “deermouse”) as the host [2], [3]. Field studies identified environmental conditions associated with increased deermouse populations and transmission in those populations, and described conditions favorable for human infection. These findings lead to predictive models [4], [5], and successful interventions to mitigate human disease [6], [7].

Through 2011, 587 HPS cases have been confirmed in the USA. The disease largely affects rural inhabitants, and has a 35% case-fatality rate (http://www.cdc.gov/hantavirus/surveillance/index.html). Numerous SNV-like viruses associated with various rodent hosts have now been identified throughout the Americas [8], [9].

In rodents, hantaviruses are primarily transmitted horizontally through biting and scratching, most frequently among male rodents [9]. Correlation analyses of field data in the SNV-deermouse system and the Seoul virus (SEOV)-*Rattus norvegicus* system revealed greater wounding frequency [10]–[12] and severity [13] in hantavirus-infected rodents. Although indirect transmission is possible among laboratory-inoculated rodents [14]–[16], it has not been observed in controlled experiments with naturally infected animals [17]. Longitudinal studies have raised questions about deermouse behavior and within-host dynamics of SNV infection and immunity in natural populations that can only be answered using controlled experiments [18].

Hantavirus rodent hosts are thought to be chronically and asymptomatically infected and shed virus for extended periods [19]. Infection in the natural host is characterized by an acute phase (7–60 days post-infection (PI)) and a persistent phase (60+ days PI). Laboratory studies have shown consistent results: after inoculation, the host experiences brief viremia 7–10 days PI. Animals develop neutralizing immunoglobulin G (IgG) 10–21 days PI, clearing virus from the blood [20], [21], but virus is sequestered in organs and adipose tissue and is continuously shed into the environment in saliva and excreta [20]. However, in a recent experiment using deermice inoculated with SNV strain SNV77734, investigators found very low titers of neutralizing antibody in infected animals and only within the first week of infection [22]. This contrasts with previous hantavirus studies including experiments using the same animal-virus model [23], which showed much higher titers of neutralizing antibody throughout the experiment. Viral RNA levels in blood have not been quantified after 21 days PI, and it is unknown if its presence coincides with viral shedding or if the quantity of viral RNA in blood is correlated with relative infectiousness. In laboratory studies, hantavirus-infected hosts show little pathology [20]; however, in field studies, SNV infection in deermice is linked to decreased survival [11], [24], [25], and decreased weight gain [26].

Botten et al., [23], [27], [28] conducted infection and transmission experiments to determine SNV viremia, transmission, and host immunological response to infection using colony-bred, wild, deermice experimentally inoculated with mouse-adapted SNV strain SN77734. They determined quantities of viral RNA in organs and tissues, corroborating some patterns seen for other Old World and New World hantaviruses [14], [16], [21], [29]–[31]. However, unlike other hantaviruses [14], [16], [21], [29]–[31], SNV was not transmitted to cage mates and not as readily isolated from saliva and excreta of experimentally infected hosts. Only 1 transmission event followed 54 attempts exposing naïve deermice to SNV77734-infected deermice [27]. Although Botten et al. [23], [27], [28] shed much light on SNV-host dynamics, questions remain about SNV transmission in deermice in nature.

Although laboratory studies of within-host transmission dynamics for hantaviruses and other microparasites provide useful information about infection and immunological processes, they are often conducted under artificial conditions. For example, caging animals in pairs eliminates population-level processes. Wild hosts undergo periods of immunosuppression due to environmental stresses, including changes in population size, breeding conditions, resource availability, and weather, which may affect transmission. Also, controlled transmission studies often rely on inoculation with a passaged virus strain [27], which may have acquired mutations impacting transmission and immunological and virological responses [32], [33]. While experimental infections enable dosage quantification and standardization, inocula differ in magnitude and delivery method from natural infections. Finally, indoor hantavirus infection and transmission studies with natural hosts require biosafety-level-4 containment.

An alternative method for investigating hantavirus-host systems is manipulative transmission experiments using naturally infected animals in outdoor enclosures. This approach eliminates emigration and immigration, but allows deermice to interact with multiple potential hosts in a familiar setting in a naturally changing environment. It allows investigators to track individual measures of aggression (wounding) and other descriptive data, and relate them to infection status and transmission cycles. By following SNV-infected deermice in a semi-controlled setting, investigators can explore possible short-term effects of infection on health that may be missed in open populations. To our knowledge, no semi-controlled outdoor hantavirus or other microparasite transmission studies using naturally infected animals have been published.

We conducted 4 transmission experiments using wild deermice in outdoor enclosures. Previously [34], we analyzed data from these studies to test hypotheses concerning ecological effects of population density and seasonality on hantavirus transmission. Here, we use molecular and immunological data to test 3 hypotheses related to SNV transmission and its potential pathologic effects on North American deermice: 1) SNV-infected deermice have a higher wound frequency than uninfected deermice; 2) deermice with higher viral RNA levels are more likely to transmit SNV, and 3) SNV-infected deermice gain less weight than uninfected deermice. We also measured antibody titers and viral RNA levels in SNV-infected hosts during acute infection and sequenced viral strains from donor deermice to investigate effects of viral strain on transmission. These hypotheses address problems within a nascent discipline we call transmission ecology, the study of within- and between-host transmission dynamics and their relationship to host population processes and environmental conditions.

## Methods

### Ethics Statement

All animal work was conducted according to the U.S. Animal Welfare Act and other relevant national and international guidelines. All components of this study were reviewed and approved by the appropriate institutional animal care and use committees (Emory University IACUC protocol #D10-1109-02R07, U.S. Centers for Disease Control and Prevention IACUC protocol #1500MILRODX-A1, and University of Montana IACUC protocol #AUP 009-07), using animal and personnel safety precautions described previously [35]. The study was also reviewed and approved under Emory University Biosafety protocol #100-2008. No trapping permit is required for trapping rodents in Montana. This study was covered under two separate protocols. In 2007, our CDC IACUC protocol called for euthanasia of the deermice at the end of the experiment; this was accomplished by overdose of inhalant anesthesia (isofluorane). In 2008, our Emory IACUC protocol required us to release the deermice back into the habitat from which they were captured following the experiment.

### Study site and experimental design

**Figure 1:**
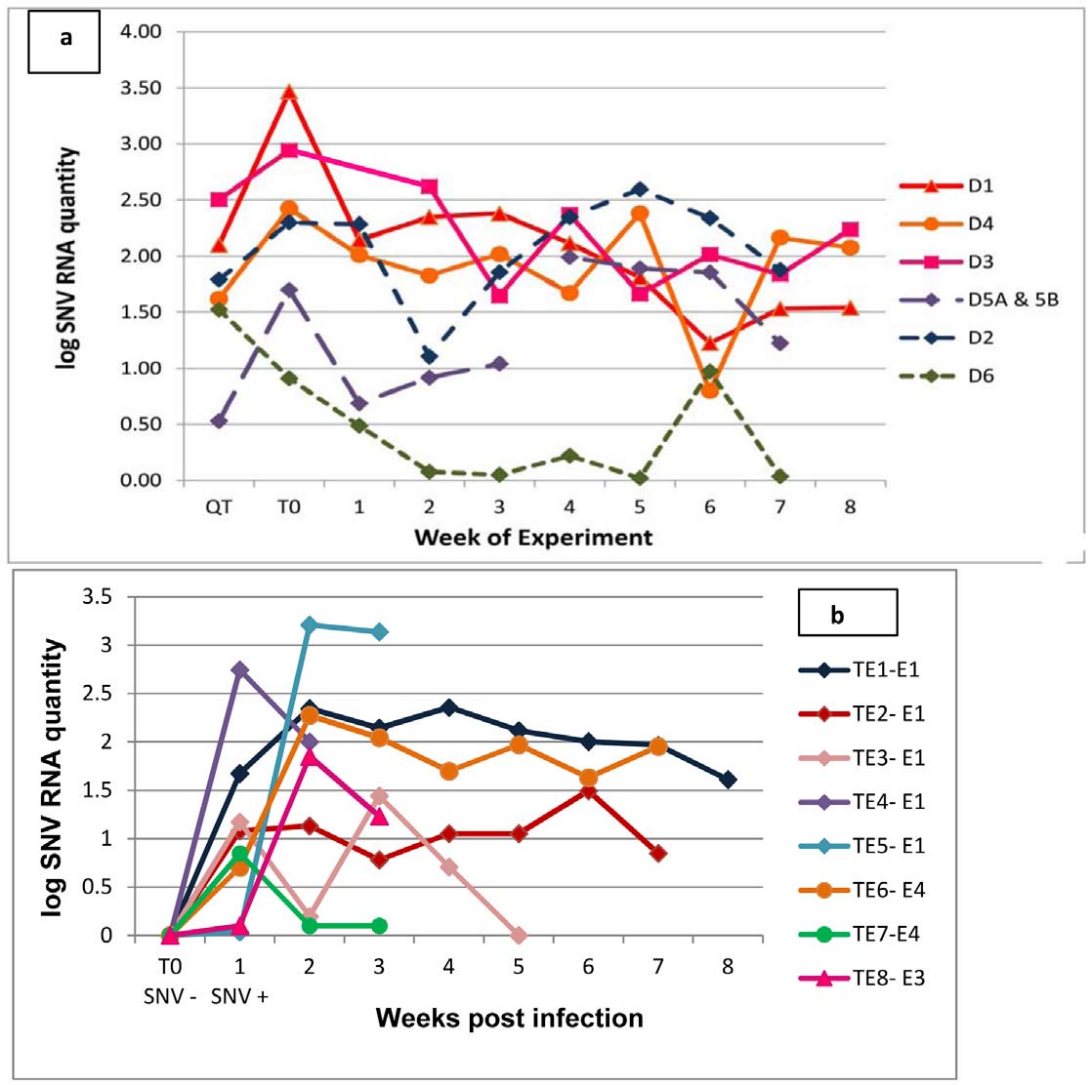
Sin Nombre hantavirus (SNV) RNA levels for (a) donor deermice before and during experiment 4, and (b) transmission event (TE) deermice at time zero (T0) and during experiment 4. a) We recovered the remains of the original donor (D5a) in enclosure 5 on week 4, and substituted a new donor (D5b). Insufficient sample was collected from donor 3 on 8/1/08 for qRT-PCR analysis. Viral RNA was quantitated from all blood samples collected starting at initial capture from the wild (QT) until the end of the experiment. b) E1, E4, and E3 are the enclosures in which each TE deermouse became infected. T0 represents the last blood sample negative for both SNV RNA and antibody to SNV before SNV RNA was first detected. Testing blood samples included retesting the initial SNV-positive sample (as indicated by prior serology or nested RT-PCR) and all subsequent blood samples, as well as blood collected at 2 or more timepoints before the initial positive test. SNV RNA quantities are proportional (see Methods), not actual copy numbers.

**Figure 2:**
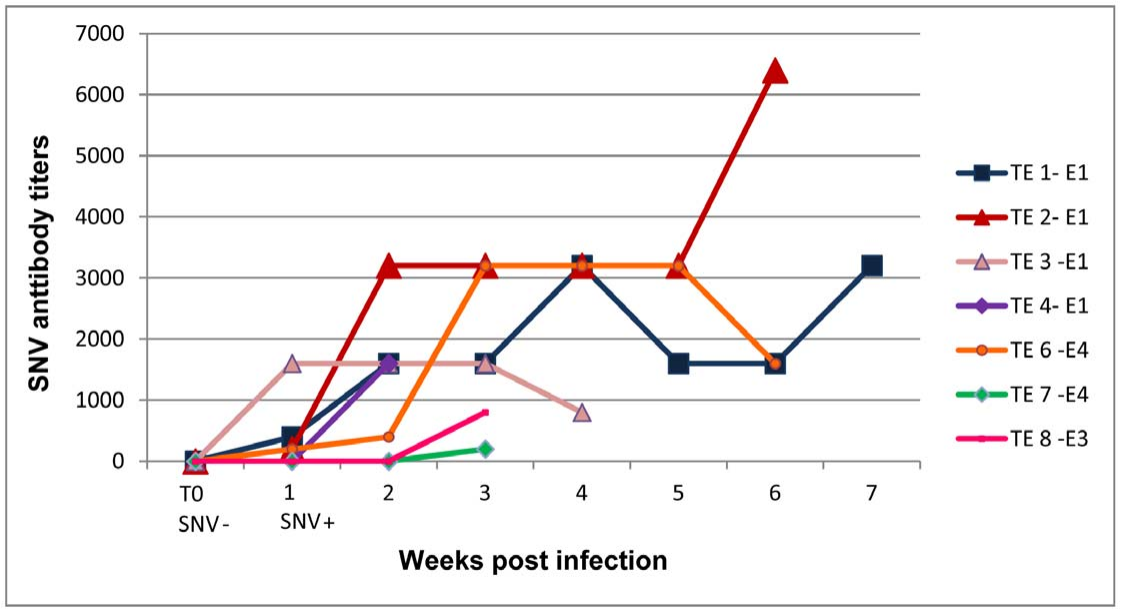
SNV antibody titers in all TE deermice in experiment 4. E1, E4, and E3 are enclosures in which each TE deermouse became infected. T0 represents the last blood sample negative for both SNV RNA and antibody to SNV before antibody or RNA was detected.

**Figure 3:**
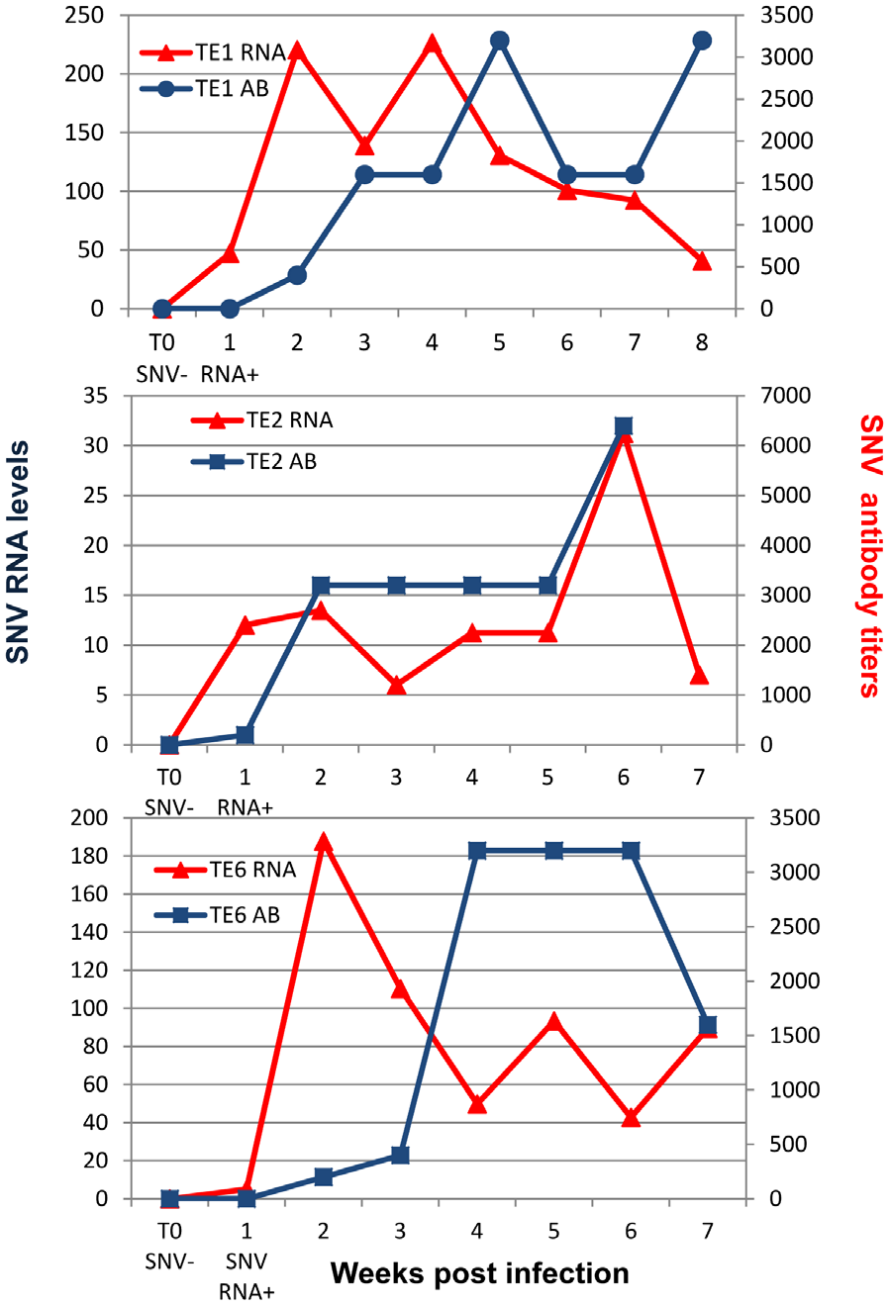
SNV RNA levels and SNV antibody titers for TE deermice with the longest time course of infection. T0 represents the last blood sample negative for both SNV RNA and SNV antibody before SNV RNA or antibody was detected.

**Figure 4:**
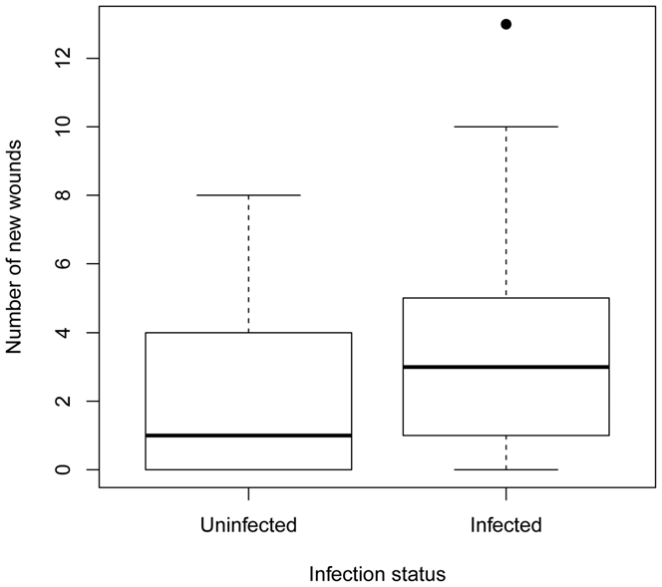
Median number of new wounds per individual deermouse by infection status. Thick horizontal line is the median; top and bottom of boxes represent the 25 and 75 percentile, respectively; horizontal lines at ends of dashed lines represent the minimum and maximum values, excluding one outlier (black dot). The infected category includes all donor and TE deermice from all 4 experiments. The uninfected category includes all susceptible deermice that never seroconverted. Each deermouse is represented only once in the analyses.

For a complete explanation of field methods, see [34]. Briefly, we conducted 4 experiments (Table 1) in 6, 0.1-ha sheet-metal enclosures [36], [37] in grassland near Butte, Montana, USA, in 2007 and 2008. All molecular and immunological data (Figures 1, 2, 3) reported are from the 2008 experiment (Table 2). Insufficient blood samples from 2007 experiments precluded analyses for the non-behavioral variables. All 4 experiments are included in the analyses of wound data (Figure 4).

**Table 1:** Experimental design for Sin Nombre hantavirus (SNV) transmission experiments in North American deermice (*Peromyscus maniculatus*) in outdoor enclosures in Montana.

EXP	Span	# Susceptible deermice per enclosure	# Donor mice per enclosure	Total # of deermice	Duration (weeks)	Sampling frequency	Transmission events
1	Jun–Jul 2007	3	1	24	4	every 2 weeks	6
2	Jul–Aug 2007	3 or 7	1	36	4.5	every 2 weeks	4
3	Sept–Oct 2007	3 or 7	1	36	6	every 2 weeks	1
4	Aug–Sept 2008	3 or 7	1	36	8	every week	8

**Table 2:** Experimental details for experiment 4.

Enclosure	Total # of deermice	Donor deermouse	# Susceptible deermice	Transmission event (TE) deermice	# TE deermice
1	8	D1	7	TE1-E1, TE2-E1,TE3-E1,TE4-E1, TE5-E1	5
2	4	D2	3	None	0
3	8	D3	7	TE8-E3	1
4	4	D4	3	TE6-E4,TE7-E4	2
5	4	D5a*, D5b	3	None	0
6	8	D6	7	None	0

* We recovered the remains of the original donor (5a) in enclosure 5 on week 4, and substituted a new donor (5b).

Deermice for enclosures were trapped within 5 km of the study area. We selected adult (>17 g) male deermice to eliminate effects of sex and age, and because adult males are responsible for most SNV transmission in wild populations [9]. One infected (donor) and a predetermined number of uninfected (susceptible) deermice were released into each enclosure according to study design (Table 1). Experimental deermice were ear-tagged with sequentially numbered metal tags. Each enclosure contained 4 underground nest burrows [38] for shelter. Nest burrows were emptied weekly during the experiments and disinfected between experiments. Rodents in enclosures were trapped weekly (2008) or biweekly (2007) by setting 36 Sherman live-capture traps in each enclosure for up to 3 consecutive nights (until all deermice were captured). Blood samples collected from the submandibular vein or retro-orbital capillary plexus of anesthetized deermice [39] were frozen on dry ice and stored at −70°C until testing for SNV antibody and SNV RNA as described previously [34], [40]–[42]. Body weight, breeding condition (scrotal or abdominal testes), trap location, and the presence and number of wounds on the tail and ear were recorded at each capture.

In 2007 (Experiments 1–3), we designated deermice as susceptible if they had no detectable SNV RNA or antibody in blood. Deermice were not quarantined prior to release into the enclosures. In 2008 (Experiment 4), potential susceptible deermice were quarantined 3 weeks in separate plastic mouse boxes in a locked quarantine facility. Deermice negative for SNV RNA and antibody were individually quarantined and retested 14–16 days and 25 days post-capture before release into enclosures. In 2007, we chose deermice positive for SNV RNA or antibody as donors. In 2008, the quarantine allowed us to choose recently seroconverting deermice, which are more likely to be infectious [21], [29], [43].

After the start of each experiment, susceptible deermice found positive for SNV RNA or antibody were designated as transmission event (TE) deermice. Because the deermice in 2007 experiments were not quarantined, it is possible that some were infected prior to release into the enclosures.

### Immunological procedures

In order to screen for SNV antibody-positive individuals and to determine SNV antibody titers in infected animals, we utilized a rapid peroxidase enzyme-linked immunosorbent assay (PAGEIA) [40]. This assay detects antibody to the nucleocapsid protein, not the Gn and Gc glycoproteins; thus a positive result does not necessarily mean that neutralizing antibody is present. As the PAGEIA utilizes a staphylococcal protein-A and streptococcal protein-G horseradish peroxidase conjugate, it has the highest affinity for IgG subclasses of multiple mammalian species, but may also bind IgM and IgA antibody [40]. Blood samples were initially diluted 1∶100 in phosphate buffered saline (PBS), and added to a 96-well polyvinyl chloride (PVC) plate that was coated with the recombinant nucleocapsid antigen in PBS and blocked 1–3 days prior to testing [40]. To determine antibody titers, the diluted 1∶100 positive samples (samples that that had an OD value 0.200 above the negative control value) were added to the first column of another antigen-coated and blocked PVC plate, then serially diluted in a log2 series from 1∶100–1∶128,000. The EIA was run as described in [22], [40]. Each plate also included a positive control and a PBS-only negative control. The sample endpoint was the dilution that had an OD value 0.100 above the negative control. The titer for a sample is reported as the reciprocal of the greatest dilution that yielded a positive result [22].

### Molecular procedures

#### RNA extraction

To prevent cross-contamination, RNA extractions were conducted in a separate laminar-flow biosafety cabinet. We handled all PCR amplicons in a separate laboratory space with equipment and supplies solely dedicated to their analyses.

Blood samples (approximately 50 uL) were added to Tripure Reagent (Roche Applied Science, Indianapolis, IN, USA) at 1∶10 and incubated for 10 min to inactivate virus. We added 250 uL of molecular grade chloroform to each sample and incubated on ice for 10 min with frequent vortexing. We centrifuged the samples at 4°C for 15 min at 12 K, removed 400 uL of the aqueous phase, and mixed it with 70% ethanol in a 1∶1 ratio. We applied the mixture to Qiagen RNAeasy columns (Qiagen Inc., Valencia, CA, USA), and followed the manufacturer's protocol until the BPE wash step. At that point, we added 500 uL RPE to the columns and centrifuged twice for 2 min to ensure removal of residual salts before continuing with the manufacturer's instructions until the final elution in 50 uL of RNAse-free H_2_O. RNA samples were stored at −70°C.

#### RT-PCR

In 2010–2011, we implemented a new RT-PCR assay to sequence viral strains from blood samples of donor and TE deermice from experiment 4. As hantaviruses have highly conserved, complementary terminal sequences [44], we sequenced the S and M segments except for the highly conserved terminal 3′ and 5′ ends (nt 22–2020 for S and nt 22–3685 for M in comparison to Convict Creek virus, Genbank Accession number (ACCN #) L33816. We used 5 uL of total RNA extracted from blood samples in RT-PCR assays with the Superscript III One-Step RT-PCR with Hi Fidelity Taq Kit (Invitrogen, Carlsbad, CA, US). The RT and cycling conditions were principally as suggested by the manufacturer; cDNA synthesis: 55°C for 30 min, pre-denaturation at 94°C for 2 min, followed by 40 cycles (45 cycles for primer set S1L/830R) of 94°C for 15 sec, 55°C (50°C for primer set S1246/2047R; see Table S1) for 30 sec, and 68°C for 1 min, and a final extension at 68°C for 5 min. PCR products were purified and sequenced using the PCR primers or internal sequencing primers (Table S1) by Beckman Coulter Genomics (Danvers, MA, USA). We used the program Primer3 to design all RT-PCR primers, except for S1L, M1L, S2047R, M3696R, which were the first and last 22 nt in the S and M segments, respectively. We performed initial sequence alignments using DNASTAR Lasergene programs Seqman and MegaAlign. All reported sequences have at least 2 sequencing passes in each region, except for the initial and final 40 nt in the highly conserved termini, which have at least 1 pass.

#### qRT-PCR

We used 5 µL of extracted total RNA from donor and TE deermouse blood samples from Experiment 4 in a qRT-PCR assay designed by PrimerDesign, Ltd. (Southampton, UK). The SNV primer-probe set targeted an 81 nt portion of the S segment, from nt 1785–1866 (in reference to Convict Creek virus ACCN # L33816), that is highly conserved across Montana SNV strains and all published SNV and Convict Creek virus strains. We used sense primer 5′-GATCTTATTGCAGCTCAGAAAYTGG-3′, antisense primer 5′-YTTTTTCCTTTARATGGTCATCAGG-3′, and probe 5′-CTGTTGGATCAACAGGTTTTGAAGCC-3′. We used glyceraldehyde 3-phosphate dehyrdogenase (GAPDH) as our endogenous control, targeting a 108 bp segment of the gene with sense primer 5′-CGGTGCCAAAAGGGTCATC-3′, antisense primer 5′-CGTTGCTGATAATCTTGAGTGAAT-3′, and probe 5′-CTTCTGCTGACGCCCCCATGTTTGTGAT-3′ (PrimerDesign, Ltd). We used Express One-Step Superscript III RT-PCR with Premixed ROX (Invitrogen). PCR-grade water was used as the negative control. The samples were cycled as suggested by Invitrogen and PrimerDesign; cDNA synthesis: 50°C for 15 min, followed by 40 cycles of 95°C for 20 sec, 95°C for 1 sec, and 60°C for 20 sec. For each sample in which SNV RNA was detected, we calculated 
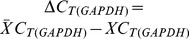
 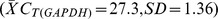. We normalized each sample by adding 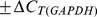 to the SNV C_T_ value. We then entered the adjusted SNV C_T_ value into the equation, 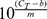 where b = y-intercept and m = slope. We used the slope and y-intercept values calculated from the standard curve to calculate the relative SNV RNA quantities for samples from that plate. All RNA samples per deermouse were run on the same plate and compared to the standard curve run simultaneously on the same plate. Because of limited RNA volumes, all samples were run in duplicate. Our standard curve consisted of serially diluted supernatants of VERO E6 cells infected with SNV strain NMR11, 10^5^–10^−1^ viral titer. Assay efficiency ranged 95–103%. Our standard curves produced correlation coefficients (r^2^) of 0.993–0.997. The RNA quantities reported are not viral copy numbers, but arbitrary units to demonstrate the fold differences in viral load over time for each deermouse.

### Statistical Analyses

We conducted statistical analyses using Microsoft Excel 2007 and R (R Development Core Team, Vienna, Austria, 2011). We used Fisher's exact two-tailed test (FET) to compare proportions of infected and uninfected deermice in relation to wounding and survival and t-tests to compare mean weight gain and wound number. To further explore the relationship between wounding and infection, we used a linear regression model with infection status as a categorical predictor variable, and number of new wounds per experiment as the outcome. The total number of new wounds was counted on an individual animal over the course of the experiment. This conservative measure only notes wounds detected on a new location on the deermouse (tail vs. ear) and increases in the number of wounds from the previous sampling session [45].

We excluded data from one donor in enclosure 3 in Experiment 1 from wound analyses because of unclear infection status. In wound and rate of weight change analyses of 2008 deermice, we excluded information from 3 deermice that were in the experiment <2 weeks. Only 5 (of 8) TE deermice from Experiment 4 had enough pre- and post-seroconversion weight data for analysis of rate of weight change in relation to seroconversion.

## Results

In 2008, 21/80 (26% of male deermice captured) were infected with SNV, as determined by antibody and RNA analyses. We chose 7 donors from the 21 SNV-infected males for use in the experiment (see methods) [34]. Although all 7 donors were from the same capture site, they yielded 3 SNV S-segment sequences (Table 3). Of the 3 successful donors, donors 1 and 3 were infected with SNV-MH1, and donor 4 with SNV-MH2 (see Fig. 1a for viral RNA loads). No differences in transmission were observed among viral substrains; SNV-MH1 and SNV-MH2 were both transmitted to all but one susceptible deermouse within their respective enclosures (Table 2). All virus sequences from TE deermice were 100% identical to those of the suspected donor. Donor deermice that successfully infected other deermice (red, orange, and pink solid lines, Fig. 1a) tended to have higher mean RNA levels in blood than unsuccessful donors (purple, blue, and green dashed lines, Fig. 1a) (successful donors n = 3, 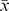 = 264.14, SD = 152.05; unsuccessful donors: n = 4, 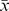 = 66.17, SD = 75.98; two sample t-test with unequal variances: t_3_ = 2.06, p = 0.065).

**Table 3:** Small (S) and medium (M) segment sequence identities at the nucleotide level for SNV variants infecting donors from experiment 4.

SNV variant	Donors infected by strain	S Segment ACCN#	S segment identity (%)		M segment ACCN#	M segment identity (%)
			SNV-MH2	SNV-MH3		SNV-MH2
SNV-MH1	1†, 2, 3†, 5b, 6	JQ690276	97.8	98.5	JQ690279	98.6
SNV-MH2	4†	JQ690277	NA	97.9	JQ690280	NA
SNV-MH3	5a	JQ690278	NA	NA	‡	NA

NA: Not applicable.

ACCN#: Genbank accession number.

† successful donor.

‡ Insufficient sample, unable to sequence.

All TE deermice experienced an initial peak in SNV RNA in blood 1–2 weeks PI (Fig. 1b). Except TE 5 from enclosure 1, all 8 TE deermice developed antibodies to SNV within 2–3 weeks PI (Fig. 2). Overall RNA levels diminished after the initial antibody response (Fig. 3; also compare Fig. 1b to Fig. 2), but spiked frequently, sometimes to or above initial peak levels (Fig. 3).

TE deermice had variable antibody titer patterns (Fig. 2), and some deermice sustained higher levels of SNV RNA in blood throughout the initial phase of infection. For example, TE 1 had 10 times more viral RNA than TE 2 during the first 4 weeks PI, even though these deermice were infected with the same virus variant (Fig. 1b and 3).

The number of new wounds per deermouse was significantly higher in infected than uninfected deermice in all experiments (t_104_ = 2.12, p = 0.04, β = −1.2533, SE = 0.5892; Fig. 4). In all experiments, no significant differences in proportion of deermice with wounds were observed between uninfected and infected deermice (FET: p = 0.30).

No significant differences were observed in weight gain or loss (g/week) between infected (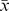 = −0.29, SD = 0.67) and uninfected (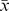 = −0.02, SD = 0.59) deermice in experiment 4 (t_35_ = −1.19, p = 0.12). Also, we saw no differences in the rate of weight change before (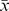 = −1.81, SD = 5.52) and after (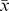 = −0.33, SD = 0.85) seroconversion in TE deermice for which this comparison was possible (paired 2-sample test for means: t_5_ = −0.61, p = 0.29), nor a significant difference between infected and uninfected deermice in the proportion that died during experiment 4 (FET: p = 0.40).

## Discussion

Our objectives included measuring SNV RNA loads and antibody titers in blood samples from naturally infected deermice during the acute phase of SNV infection, and determining the influence of viral RNA load and viral strain on SNV transmission. We also tested whether SNV-infected deermice were more likely to be wounded and accrue more wounds than uninfected mice. These objectives are critical to understanding the natural cycle of infection in an individual host, but have not been explored using serially collected samples from naturally infected individuals over time and related to population-level processes. We also explored host weight changes and survival in relation to SNV infection.

Five of 8 TE deermice developed peak SNV RNA levels in blood 2 weeks after their last RNA-negative result, while 3 developed peak levels 1, 3, and 6 weeks after their last negative result. Our field results are similar to those of a laboratory experiment [21] in which cotton rats experimentally infected with Black Creek Canal virus developed peak infectious virus titers in blood at 14 days PI. By sampling weekly, we demonstrated viral RNA in blood for at least 8 weeks PI (throughout the acute phase of infection). These findings are in direct contrast to previous studies indicating hantaviruses are cleared from blood 10–21 days PI [20], [21], and then only intermittently detected [21], but reinforces a recent study showing viral RNA in blood at various timepoints during the acute and persistent phases [28]. Although some investigators did not find hantavirus RNA in blood throughout the acute phase, they found that rodents shed infectious virus [21] or viral RNA [16] past 10–21 days PI in saliva and excreta. Also, a recent study showed that T-cells isolated from deermice with experimental SNV infections include components of immunosuppressive regulatory T-cell activity (expressing Forkhead box P3 transcription factor) and cytokines (TGF-β_1_ and IL-10) associated with downregulating inflammatory responses [46]. Such discoveries for New [46] and Old [47] World hantaviruses indicate that hantavirus infection diminishes the adaptive immunological response, allowing the virus to be maintained in the host's blood during the acute phase of infection and permitting the virus to establish a persistent infection within the host.

Most laboratory studies euthanize experimental animals at predetermined intervals. By sampling the same animals for up to 8 weeks PI, we showed that viral RNA levels and anti-SNV antibody titers varied highly over time, even within an individual. Peak viral RNA levels varied greatly in TE deermice 1–2 weeks PI and, after the antibody response, RNA levels in blood changed differently (Figs. 2 & 3). Our results corroborate a recent experiment showing highly variable SNV viral RNA levels in lung and heart tissue and variable SNV antibody levels among experimentally infected deermice in the initial 20 days PI [22]. Variable immune responses to infection are common in wild outbred deermice [22], [28]. Our wild deermice were also exposed to environmental stressors, which can affect immune responses and viral RNA levels.

The 3 donor deermice that infected other deermice within their enclosures in 2008 tended to have higher mean SNV RNA levels over the experiment (p = 0.065). Although unsuccessful donors 5a, 5b, and 6 maintained lower RNA levels (Fig. 1a), donor 2 had increased viral load near the end of the experiment and may have infected other deermice that were not detected before the conclusion of the experiment. These data are suggestive, but additional studies are needed to clearly determine whether a threshold SNV RNA level prompts SNV transmission. Because of the limited sample size, the power of our statistical comparison was low.

That the viral strains transmitted to TE deermice were identical to one another and to the donor deermouse strain within each enclosure indicates that all TE deermice in an enclosure were infected by the donor's strain, either directly by the donor or by another deermouse infected by that donor. These molecular data corroborate our trapping data, showing no non-experimental deermice entered the enclosures during experiment 4 and populations within the enclosures were effectively closed. Although we know, within 1 week, the time each deermouse became infected, after the first transmission case, molecular data do not indicate which deermice propagated the infection. Future studies could implement cameras, pit tag recorders, and fluorescent marking powder [48] to identify contact structures and their relationship to the chain of infection within enclosed deermouse populations.

The finding that infected deermice had significantly more new wounds than susceptible deermice that never seroconverted supports studies reporting higher wound frequency [10], [11] or severity [13] in antibody-positive hosts. Because SNV is horizontally transmitted, older deermice are more likely to be infected and, because of accumulated experience, are more likely to have scars. Thus, in a random field sample, a correlation between scars and infection status is expected because both variables correlate with age. Because we chose deermice of similar age and counted only new wounds incurred during the experiment, we clearly demonstrate an association between wounding and infection while controlling for age and experience. Although the simplest explanation for this association is that infection is a consequence of aggression, aggression may also be a consequence of infection. Indeed, SEOV infection may influence host aggression [13], [49], [50]. As we saw no evidence of indirect SNV transmission, our results support the consensus that SNV is mainly transmitted directly through aggressive encounters [10], [11], [13], [39].

The nest burrows in our enclosures would be an ideal environment for indirect transmission. We found urine or feces in 75–100% of nestboxes each week. In 2007, we observed 1 donor deermouse cohabitating with the same susceptible deermouse twice and other donor deermice cohabitating with multiple deermice at least twice. None of these susceptible deermice seroconverted during the experiment. In an Andes hantavirus transmission study using naturally infected donor rodents, 16 of 130 direct transmission attempts, but 0 of 62 indirect transmission attempts were successful [17]. Previous reports of indirect hantavirus transmission [14]–[16] were conducted using laboratory inoculated hosts. Naturally infected rodents may shed less virus than experimentally infected individuals, or exposure to environmental elements outdoors may disperse or inactivate infectious virus and limit indirect transmission in the wild. Weekly cleaning of nest burrows may have also decreased the likelihood of indirect transmission. However, we cannot rule out the possibility of indirect transmission, or a mixture of indirect and direct transmission, in our experiments.

We saw no influence of viral strain on transmission; SNV-MH1 and SNV-MH2 were both transmitted to all but one susceptible deermouse within their respective enclosures. Further research is needed to determine how host genetics and other immune system components respond to SNV infection and affect virus propagation in individuals and populations.

Although other studies of Montana deermouse populations indicated SNV infection affects survivorship or weight gain, we found no statistically significant effects. This could be because we provided supplemental food and water. Also, our longest running experiment was only 8 weeks; a longer experiment might detect deleterious effects. We saw no differences in weight gain in seroconverting deermice, but had data from only 5 individuals (compared to 1,466 in a longitudinal field study) [26]. However, in TE deermouse 5 from enclosure 1 (Fig. 1b), viral RNA increased 1000-fold within 3 weeks (viral RNA levels 2–100 times higher than in other TE deermice), and no antibody response was detected before the animal's death. This single observation could have many possible explanations. However, when considered in light of recent analyses of a 15-year mark-recapture dataset from Montana showing that infected male deermice had 13.4% lower apparent survival than uninfected males and females [25], it suggests that it is possible that some deermice may not tolerate SNV infection and quickly die without being detected in mark-recapture studies that sample less frequently. Additional studies, including replicated laboratory and enclosure studies, are needed.

To estimate relative infectiousness, we assumed that viral RNA in blood indicates infectious virus. This is likely, but has not been demonstrated (e.g., RNA could be bound in noninfectious antigen-antibody complexes). In addition, we did not measure viral RNA shedding in saliva and excreta, possibly a more accurate predictor of relative infectiousness. Future studies using similar experiments to quantify viral RNA in excreta and saliva would be useful to measure virus shedding and verify whether viral RNA in blood is an accurate predictor of virus shedding.

By exploring immunological and virological components of hantavirus infection in naturally infected deermice in relation to host behavior, we provide a step toward better understanding hantavirus-host infection dynamics in the wild and broadening our understanding of rodent-borne zoonotic viruses. By clarifying the influence of ecological, behavioral, and within-host infection factors, and their interactions on infection prevalence, our research contributes to understanding the transmission ecology of SNV and other zoonotic pathogens. An example of applying the One Health concept, we combined methods and expertise from ecology, molecular biology, virology, immunology, and mammalogy. Knowledge of SNV transmission in its host populations will contribute to development of more accurate models of changing risk to humans and may lead to more effective disease prevention and mitigation at the wildlife-human interface.

## Supporting Information

Table S1 **Information on primers and reference sequences used to sequence small (S) and medium (M) segments of SNV-MH strains 1, 2, and 3.** Includes amplification regions of each primer set and reference strains used to design primers. ACCN#: Genbank Accession number.
(DOCX)
