# Supplementary material for: Transmission Ecology of Sin Nombre Hantavirus in Naturally Infected North American Deermouse Populations in Outdoor Enclosures
Source: PLoS One. 2012 Oct 26;7(10):e47731. doi: 10.1371/journal.pone.0047731 (PMC3482230; doi:10.1371/journal.pone.0047731)
Supplement: Table S1 — Information on primers and reference sequences used to sequence small (S) and medium (M) segments of SNV-MH strains 1, 2, and 3. Includes amplification regions of each primer set and reference strains used to design primers. ACCN#: Genbank Accession number. (DOCX) [file pone.0047731.s001.docx]

**Table S1. Information on primers and reference sequences used to sequence small (S) and medium (M) segments of SNV-MH strains 1, 2, and 3**. Includes amplification regions of each primer set and reference strains used to design primers. ACCN #: Genbank Accession number.

| **S Segment** |  |  |  |  |  |  |
| --- | --- | --- | --- | --- | --- | --- |
| **Amplification Region** | **Primer** | **Reference strain (ACCN#)** | **Forward primer (5’-3’)** | **Primer** | **Reference strain (ACCN#)** | **Reverse Primer** |
| nt 22-830 | S1L | Convict Creek  (L33816) | TAGTAGTAGACTCCTTGAGAAG | S830R | MtPeS09  (JQ690282) | ATTGGCGTGTTATGAAATAGGC |
| nt 632-1995 | S632L | MtPeS09  (JQ690282) | AGGTTCCGTACAATTGCCTGTG | S1995R | MtPeS09  (JQ690282) | TTCGTTGAGGTAATAGGGAAGG |
| nt 1246-2047 | S1246L | Convict Creek (L33816) | GATCCTGAACTCAGGGAACTTG | S2047R | Convict Creek (L33816) | TAGTAGTATACGCCTTGAAAAGC |
| **M Segment** |  |  |  |  |  |  |
| **Amplification Region** | **Primer** | **Reference strain (ACCN#)** | **Forward primer (5’-3’)** | **Primer** | **Reference strain (ACCN#)** | **Reverse Primer (5’-3’)** |
| nt 22-962 | M1L | Convict Creek (L33684) | TAGTAGTAGACTCCGCACGAAG | M962R | Convict Creek (L33684) | GCAGTAACAGGTCCAGCTATTC |
| nt 860-1684 | M860L | Convict Creek (L33684) | GGTTTTAAATCCAAGAGGTGAAG | M1684R | MtPo09  (JQ690284) | CAAATGTCACACACCATTGAGC |
| nt 1124-2051 | M1124L | MtPo09  (JQ690284) | AAACAGTACCCCTCACATGGAC | M2051R | MtPeS09  JQ690283 | CATGTGCTGTATCAGACCAACC |
| nt 1772-2648 | M1772L | MtPo09  (JQ690284) | GCCCATACTGCATGACAATAAC | M2648R | MtPo09  (JQ690284) | TAATCCCACCTTGCTCTAAAGG |
| nt 2364-3696 | M2364L | MtPo09  (JQ690284) | ACCAGATTGCCCAGGGGTAG | M3696R | Convict Creek (L33684) | TAGTAGTAGACTCCGCGGGAAC |
